# Effects of chrysin loaded self‐nano emulsifying drug delivery system for the treatment of Alzheimer’s disease

**DOI:** 10.1002/alz.085599

**Published:** 2025-01-09

**Authors:** Sukriti Vishwas, Sachin Kumar Singh, Monica Gulati, Kamal Dua, Violina Kakoty, Bushra Bashir

**Affiliations:** ^1^ School of Pharmaceutical Sciences, Lovely Professional University, Jalandhar, Punjab India; ^2^ Faculty of Health, Australian Research Centre in Complementary and Integrative Medicine, University of Technology Sydney, Ultimo, NSW, 2007, NSW Australia; ^3^ Discipline of Pharmacy, Graduate School of Health, University of Technology Sydney, Ultimo, NSW, 2007, NSW Australia

## Abstract

**Background:**

Alzheimer’s disease (AD) is a type of degenerative disorder that affects the brain. There are various herbal drugs that have been tested for their effectiveness in treating AD, and chrysin is one of them. Chrysin is a polyphenolic flavonoid that has several neuroprotective effects, including reducing the levels of AChE enzyme, accumulated amyloid β, oxidative stress, and neuroinflammation. It is also inherently senolytic, which helps to lessen the impact of cellular aging. However, chrysin has a limited ability to dissolve and is not easily absorbed by the blood‐brain barrier, which can reduce its effectiveness.

**Method:**

We have developed a self‐emulsifying drug delivery system (SNEDDS) for chrysin to address these issues using a Box‐Behnken design (BBD) approach. The pharmacokinetic studies showed that chrysin SNEDDS had significantly higher bioavailability than regular chrysin. The pharmacodynamic studies assessed cognitive and motor functions in rats.

**Result:**

SNEDDS has many advantages, including increased drug loading, ease of preparation, high stability, and increased bioavailability and blood‐brain barrier permeability. The optimized formulation of chrysin‐loaded SNEDDS resulted in a small droplet size, high drug loading, and good stability. The formulation was evaluated for its bioavailability, availability in the brain, and pharmacodynamic efficacy.

**Conclusion:**

The results showed that chrysin SNEDDS loaded significantly improved cognitive functions in AD‐induced rats at both low and high doses. The biochemical studies also demonstrated that chrysin SNEDDS loaded reduced the levels of AChE enzyme, amyloid β, oxidative stress, and neuroinflammation.

